# Effects of Dietary L-Theanine on Growth Performance, Antioxidation, Meat Quality, and Intestinal Microflora in White Feather Broilers With Acute Oxidative Stress

**DOI:** 10.3389/fvets.2022.889485

**Published:** 2022-06-24

**Authors:** Zixi Wang, Yanfang Tang, Lina Long, Huihua Zhang

**Affiliations:** School of Life Science and Engineering, Foshan University, Foshan, China

**Keywords:** L-theanine, intestinal flora, oxidative stress, growth performance, broiler chicken

## Abstract

In order to reduce the negative effects caused by oxidative stress on broilers, it is particularly important to find ways to alleviate oxidative stress. As a natural plant extract, L-theanine has a variety of biological effects, such as improving antioxidant capacity, promoting growth, and enhancing immunity and antitumor. This trial evaluated the effects of dietary supplementation of L-theanine on growth performance, antioxidation, meat quality, and intestinal microflora in 817 White Feather Broilers. A total of 108 21-day-old 817 broilers with similar body weight (BW) were randomly divided into three groups with six replicates per group and six chickens within each replicate. The three groups were corn-soybean-based diet (NC group); basal diet plus drinking water with 30 mg hydrocortisone/kg (PC group); and basal diet supplemented with 400 mg L-theanine/kg plus drinking water with 30 mg hydrocortisone/kg (LT group). Compared with the NC group, from 21 to 24 days of age, the PC and LT groups had decreased BW, average daily gain (ADG), and average daily feed intake (ADFI), and increased feed to gain ratio (F/G; *p* < 0.05). At 24 days of age, the LT group had improved superoxide dismutase (SOD) and glutathione peroxidase (GSH-Px) activities in serum as compared to the NC group (*p* < 0.05). The LT group broilers also had significantly higher concentrations of malondialdehyde (MDA) in serum and liver (*p* < 0.05). On the 42nd days, the PC group had lower PH_45min_ (*p* < 0.05) than the NC and LT groups and higher cooking loss and shear force (*p* < 0.05). Moreover, the villi height of the PC group was significantly lower in jejunum than the NC group (*p* < 0.05). The LT group had a higher ZO-1 content in duodenum than the NC and PC groups (*p* < 0.05). The activity of GSH-Px in the liver of the LT group was increased than in the PC group (*p* < 0.05). The relative abundance of *Firmicutes* in the LT group was significantly higher than in the NC and PC groups (*p* < 0.05). These results suggested that the effects of acute oxidative stress on growth performance and meat quality of broilers are continuous, and dietary supplementation of L-theanine could improve the growth performance and meat quality, enhance the intestinal mucosal barrier and antioxidant capacity, and improve the composition of the intestinal flora of broilers caused by acute oxidative stress.

## Introduction

Oxidative stress is an imbalance between the production of cell-damaging free radicals and the body's ability to neutralize them (1, 2). Oxidative stress exerts a series of adverse effects on broiler breeding, such as decreased immunity, feed intake, quality of broilers, and slower daily weight gain (3–5). For the moment, many places around the world, such as China, have forbidden to add antibiotic growth promoters in poultry diets. Therefore, it is imperative to find an effective, safe, and sustainable natural additive that can both increase the productive potential and maintain broiler health.

Previous studies have demonstrated the great potential of plant-extracted natural polysaccharides as an alternative to antibiotic additives (6, 7). L-theanine is a characteristic non-protein amino acid extracted from the tea. It is one of the flavor substances of tea, accounting for 1–2% of the weight of the dry tea, with a chemical formula C_7_H_14_N_2_O_3_. Studies have found that L-theanine has various biological activities, such as antioxidant, promoting animal growth, enhancing immunity, and protecting nerves (8, 9). Intestinal states, such as intestinal mucosal morphology, barrier integrity, and flora, are closely related to animal growth and health status (10). The intestine is an important organ for absorbing nutrients and digesting. In the state of oxidative stress, excessive accumulation of free radicals destroys the intestinal barrier. The damage in the intestine is easy to cause intestinal absorption dysfunction, bacterial community disorder, and decreased immunity (11, 12). The purpose of this experiment was to study the effects of a low dosage of L-theanine have on growth performance, antioxidant capacity, meat quality, and intestinal states of 817 White Feather Broilers that subjected to hydrocortisone-induced acute oxidative stress. It can provide theoretical guidance for practical production.

## Materials and Methods

### Experimental Design, Animals, and Housing

This study was approved by the Ethics Committee of Foshan University (Fosu2022024), China. All experimental procedures involving the use of animals were conducted in compliance with relevant laws and institutional guidelines. A total of 108 21-day-old 817 broilers (a hybrid of high-quality male broiler and female laying hens) with similar body weight were randomly divided into three groups with six replicates/cages per group and six chickens within each replicate (obtained from Guangdong Muyuan Ltd, China). The three groups were corn-soybean-based diet (NC group); basal diet plus drinking water with 30 mg hydrocortisone/kg (PC group); and basal diet supplemented with 400 mg L-theanine/kg plus drinking water with 30 mg hydrocortisone/kg (LT group). All chickens were raised in a wire cage of 65 × 65 × 42 cm, in an environmentally controlled room with continuous light and *ad libitum* access to feed and water throughout the 21 days experiment.

Hydrocortisone is a glucocorticoid. Previous studies showed that it can induce oxidative stress in rats (13). The preliminary study demonstrated that the hydrocortisone can also induce oxidative stress in broilers. Chemical formulas of hydrocortisone and L-theanine (Aladdin Biochemical Technology Co., Ltd., Shanghai, China) respectively are C_21_H_30_O_5_ and C_7_H_14_O_3_, both have a purity of 98%.

All diets were formulated to meet the National Research Council (NRC, 1994) nutrient requirements. The ingredients and chemical composition of the basal diet are shown in Table 1.

**Table 1:** Composition and nutrient content of experimental diets.

	**Basal diets**	
	**Starter (21–27 days, age)**	**Finisher (28–42 days, age)**
**Ingredients g/kg**		
Corn	58.49	57.65
Soybean meal(43%CP)	29.08	27.21
Corn gluten meal(60%CP)	5.00	5.00
Limestone	1.36	1.22
Calcium hydrogen phosphate (16.5%)	1.00	1.04
L-lysine Sulfate (70%)	0.49	0.39
DL-methionine (98.5%)	0.27	0.21
NaCl	0.32	0.32
Montmorillonite	0.20	0.20
L-Threonine	0.10	0.06
Mineral Premix	0.13	0.13
Choline chloride (50%)	0.08	0.08
Vitamin Premix	0.03	0.03
Phytase (20,000IU)	0.01	0.01
Sodium humate	0.10	0.15
Lard	3.34	6.30
Total (%)	100	100
**Nutrient content**		
ME (Kcal)	2796	2974
CP (g/kg)	20.62	19.61
Ca (g/kg)	0.85	0.80
Total P (%)	0.55	0.54
Available P (g/kg)	0.30	0.30
Lys (%)	1.34	1.21
Met (%)	0.58	0.52

*Supplied per kilogram of diet: vitamin A (trans-retinyl acetate), 10,050IU; vitamin D3, 2,800 IU; vitamin E (DL-a-tocopheryl acetate), 50 mg; vitamin K3, 3.5 mg; thiamine, 2.5 mg; riboflavin, 7.5 mg; pantothenic acid, 15.3 mg; pyridoxine, 4.3 mg; vitamin B12 (cyanocobalamin), 0.02 mg; niacin, 35 mg; choline chloride, 1,000 mg; biotin, 0.20 mg; folic acid, 1.2 mg; Mn, 100 mg; Fe, 85 mg; Zn, 60 mg; Cu, 9.6 mg; I, 0.30 mg; Co, 0.20 mg; and Se, 0.20 mg*.

### Growth Performance

All broilers were individually weighted before the experiment, on day 3, and on day 21 after a 12 h fast. The feed intake of broilers in each replicate was recorded to determine average daily gain (ADG) and average daily feed intake (ADFI). The F/G was calculated by ADFI/ADG.

### Sample Collection

On day 3 and 21, one broiler was randomly selected from each replicate and marked. After 12 h of fasting, blood samples (3–5 ml) were collected from the wing vein. The sample was allowed to clot at 37°C for 2 h and subsequently centrifuged at 3,500 rpm for 15 min at 4°C to obtain serum and stored at −80°C refrigerator for future testing. The broiler was slaughtered by cervical dislocation after bleeding on day 21 of the experiment. Liver tissue samples were harvested, stored at −80°C, and used for antioxidant capacity assays. Then the right side of the pectoralis was collected to measure muscle PH and meat quality. The mucosa of the duodenum, jejunum, ileum, and the middle intestinal segment was collected and then preserved with 4% formaldehyde phosphate buffer saline (PBS) solution. The contents of the cecum and duodenum were collected and stored at −80°C for later testing.

### Assay of Antioxidant Indices

The liver was isolated on an ice tray and homogenized with cold saline with a weight-to-volume ratio of 1:9. The homogenate was centrifuged at 9,000 × g for 10 min at 4°C. The supernatant was collected for measuring the indices. The total antioxidant capacity (T-AOC), glutathione peroxidase (GSH-Px), superoxide dismutase (SOD), and malondialdehyde (MDA) concentration were determined with commercially assay kits (Nanjing Jiancheng Bioengineering Institute, Nanjing, China) according to the method described by Yan et al. (14) and Wen et al. (15).

### Small Intestinal Morphology

The intestinal tissue samples were embedded in paraffin-wax firstly, and three consecutive sects (5 μm) were stained with hematoxylin-eosin for analysis. Villi height and crypt depth were measured by using a microscope (MD50-T, OLYMPUS, China) at 40 × magnification. A total of 20–30 villi and their associated crypts per section were randomly selected, and the villus length and crypt depth were analyzed by MShot Image Analysis System. Three different intestinal villi and crypts were measured in each sample. The intestinal tight junction protein ZO-1 was measured by using a ZO-1 enzyme-linked immunosorbent assay (ELISA) kit (Nanjing Jiancheng Institute of Bioengineering, Nanjing, China) in accordance with the manufacture's instructions.

### Intestinal Flora Analysis

Total genome DNA was extracted from samples using cetyltrimethylammonium bromide (CTAB)/sodium dodecyl sulfate (SDS) method. DNA concentration and purity were monitored on 1% agarose gels. DNA was diluted to l μg/μl using sterile water. 16S rRNA genes of distinct regions (V3–V4) were amplified using a specific primer with the barcode. All PCR reactions were carried out with 15 μl of Phusion® High-Fidelity PCR Master Mix (New England Biolabs); 0.2 μM of forward and reverse primers and about 10 ng template DNA. Thermal cycling consisted of initial denaturation at 98°C for 1 min, followed by 30 cycles of denaturation at 98°C for 10 s, annealing at 50°C for 30 s, elongation at 72°C for 30 s, and finally, 72°C for 5 min. Sequencing libraries were generated using TruSeq® DNA PCR-Free Sample Preparation Kit (Illumina, United States) following the recommendations of manufacturer, and index codes were added. The library quality was assessed on the Qubit@2.0 Fluorometer (Thermo Scientific) and Agilent Bioanalyzer 2100 system. At last, the library was sequenced on an Illumina NovaSeq platform and 250 bp paired-end reads were generated.

### Statistical Analysis

All data were analyzed using the one-way ANOVA of SPSS 20.0 (SPSS Inc., Chicago, IL, United States). The data were analyzed as a completely randomized design with the cages as the experimental unit. Orthogonal polynomial contrasts were used to determine linear and quadratic effects of the increasing L-theanine. Statistical significance was declared at *p* < 0.05 and trends at 0.05 < *p* < 0.10.

## Results

### Growth Performance

The effects of dietary L-theanine supplementation on the growth performance of broilers under the oxidative stress are shown in Table 2. From 21–24 to 21–42 days of age, when compared with the NC group, the PC and LT groups had significantly lower BW, ADG, and ADFI (*p* < 0.05) and higher F/G at the 21–24 days (*p* < 0.05). In addition, the F/G in the PC group was higher than in the other two groups at 21–42 days (*p* < 0.05).

**Table 2:** Effects of dietary L-theanine on the performance of broilers under the oxidative stress^1^.

**Items**	**Groups**			***p*** **-value**
	**NC^3^**	**PC^3^**	**LT^3^**	
Initial weight (21 days, g)	441.40 ± 7.68	441.08 ± 7.45	441.62 ± 8.83	0.915
**21–24 days, age**				
BW, g	558.89 ± 8.61^a^	478.89 ± 5.09^b^	473.33 ± 8.82^b^	<0.001
ADG, g	39.07 ± 2.87^a^	12.41 ± 1.70^b^	10.55 ± 2.94^b^	<0.001
ADFI, g	104.95 ± 6.67^a^	77.64 ± 2.53^b^	72.20 ± 4.18^b^	<0.001
F/G^2^	2.70 ± 0.30^a^	6.33 ± 0.74^b^	7.14 ± 1.63^b^	<0.001
**21–42 days, age**				
BW, g	1392.50 ± 49.07^a^	974.67 ± 70.47^b^	1060.00 ± 85.14^b^	<0.001
ADG, g	45.36 ± 2.34^a^	25.46 ± 3.35^b^	29.52 ± 4.05^b^	<0.001
ADFI, g	102.96 ± 12.73^a^	67.92 ± 3.02^b^	65.98 ± 9.47^b^	0.002
F/G	2.27 ± 0.20^a^	2.69 ± 0.29^b^	2.23 ± 0.15^a^	0.039

a, b *Means within a row with different superscripts differ significantly (P < 0.05)*.

1 *Data represent the means of six broilers for one broiler per replicate (n = 6)*.

2 *F/G, feed to gain ratio*.

3 *PC, added hydrocortisone 30mg/kg to drinking water in broilers basal diet; LT, fed diets supplemented with 400 mg/kg L-theanine to basal diets; NC, fed a corn-soybean meal basal diet*.

### Analysis of Meat Quality

Table 3 shows the results on the meat quality of the broilers, at 42 days of age, the PH45_min_ of the PC group is significantly lower than the NC and LT groups (*p* < 0.05). The cooking loss and shear force of the PC group were higher than the NC and LT groups (*p* < 0.05).

**Table 3:** Effects of dietary L-theanine on meat quality of broilers under the oxidative stress^1^.

**Items**	**Groups**			**SEM**	***p*** **-value**
	**NC^2^**	**PC^2^**	**LT^2^**		
42 days, age					
PH_45min_	6.69^a^	6.36^b^	6.83^a^	0.040	0.009
Cooking loss, %	7.70^a^	9.63^b^	8.18^a^	0.194	0.003
Shear force, *N*	7.23^a^	9.61^b^	7.89^a^	0.270	0.006

a, b *Means within a row with different superscripts differ significantly (p < 0.05)*.

1 *Data represent the means of six broilers for one broiler per replicate (n = 6)*.

2 *PC, added hydrocortisone 30mg/kg to drinking water in broilers basal diet; LT, fed diets supplemented with 400 mg/kg L-theanine to basal diets; NC, fed a corn-soybean meal basal diet*.

### Small Intestinal Morphology Analysis

As shown in Table 4, when compared with the NC group, the villi height of jejunum in the PC group is significantly decreased (*p* < 0.05).

**Table 4:** Effects of dietary L-theanine on intestinal morphology of broilers under the oxidative stress^1^.

**Items**	**Groups**			**SEM**	***p*** **-value**
	**NC^2^**	**PC^2^**	**LT^2^**		
**42 days, age**					
**Villus height, μm**					
Jejunum	593.40^a^	495.47^b^	559.82^ab^	11.510	0.013
Ileum	421.20	455.42	430.23	17.515	0.464
Duodenum	970.87	963.28	979.16	41.401	0.884
**Crypt depth, μm**					
Jejunum	79.70	77.61	67.64	4.565	0.329
Ileum	62.29	68.85	58.41	3.422	0.256
Duodenum	97.59	105.02	95.29	5.561	0.510

a, b *Means within a row with different superscripts differ significantly (p < 0.05)*.

1 *Data represent the means of six broilers for one broiler per replicate (n = 6)*.

2 *PC, added hydrocortisone 30mg/kg to drinking water in broilers basal diet; LT, fed diets supplemented with 400 mg/kg L-theanine to basal diets; NC, fed a corn-soybean meal basal diet*.

### ZO-1 Protein and Antioxidant Indices

At 42 days of age, the content of ZO-1 in duodenum of the LT group was higher (*p* < 0.05) than in the other two groups from Table 5.

**Table 5:** Effects of dietary L-theanine on ZO-1 protein of broilers under the oxidative stress^1^.

**Items**	**Groups**			**SEM**	***p*** **-value**
	**NC^2^**	**PC^2^**	**LT^2^**		
**42 days, age**					
Jejunum, ng/ml	5.52	5.47	5.01	0.297	0.527
Ileum, ng/ml	4.69	4.09	5.20	0.460	0.371
Duodenum, ng/ml	3.87^b^	3.80^b^	5.66^a^	0.284	0.043

a, b *Means within a row with different superscripts differ significantly (p < 0.05)*.

1 *Data represent the means of six broilers for one broiler per replicate (n = 6)*.

2 *PC, added hydrocortisone 30mg/kg to drinking water in broilers basal diet; LT, fed diets supplemented with 400 mg/kg L-theanine to basal diets; NC, fed a corn-soybean meal basal diet*.

At 24 days of age, when compared to the NC group, the Table 6 result showed that the MDA concentrations in serum and liver were significantly increased in the PC and LT groups (*p* < 0.05). In addition, the LT group had increased serum SOD and GSH-Px activities (*p* < 0.05). At 42 days of age, the activity of GSH-Px in LT was higher than in the PC group (*p* < 0.05).

**Table 6:** Effects of dietary L-theanine on antioxidant indices in the serumand hepatic of broilers under the oxidative stress^1^.

**Items**	**Groups**			**SEM**	***p*** **-value**
	**NC^2^**	**PC^2^**	**LT^2^**		
**24 days, age Serum**					
T-AOC^3^, m/M	4.71	4.48	4.38	0.095	0.204
GSH-Px^3^, U/mL	1799.79^a^	2439.03^b^	2488.55^b^	38.833	<0.001
SOD^3^, U/mL	31.54^b^	36.85^ab^	39.93^a^	1.016	0.004
MDA^3^, nmol/mL	3.11^a^	4.91^b^	4.81^b^	0.176	0.004
**Hepatic**					
T-AOC, mmol/gprot	3.51^ab^	3.71^a^	3.30^b^	0.036	0.002
GSH-Px, U/mg	133.33	124.19	120.43	4.383	0.275
SOD„U/mgprot	801.59	829.34	801.82	9.112	0.259
MDA, nmol/mgprot	1.02^a^	1.83^b^	1.97^b^	0.114	0.027
**42 days, age Serum**					
T-AOC, m/M	4.50	4.37	4.47	0.079	0.530
GSH-Px, U/mL	1635.69	1701.61	1750.35	41.532	0.311
SOD,U/mL	32.57	32.86	31.11	1.326	0.619
MDA, nmol/mL	4.02	2.98	3.59	0.217	0.100
**Hepatic**					
T-AOC, mmol/gprot	3.63	3.40	3.44	0.076	0.270
GSH-Px, U/mg	149.06^ab^	128.42^a^	178.84^b^	8.100	0.020
SOD,U/mgprot	774.35	737.98	731.20	28.298	0.564
MDA, nmol/mgprot	1.58	1.70	2.20	0.170	0.202

a, b *Means within a row with different superscripts differ significantly (p < 0.05)*.

1 *Data represent the means of 6 broilers for 1 broiler per replicate (n =6)*.

2 *PC, added hydrocortisone 30mg/kg to drinking water in broilers basal diet; LT, fed diets supplemented with 400 mg/kg L-theanine to basal diets; NC, fed a corn-soybean meal basal diet*.

3 *MDA, malondialdehyde; SOD, superoxide dismutase; GSH-PX, glutathione peroxidase; T-AOC, total antioxidant capacity*.

### Intestinal Flora

**Figure 1:**
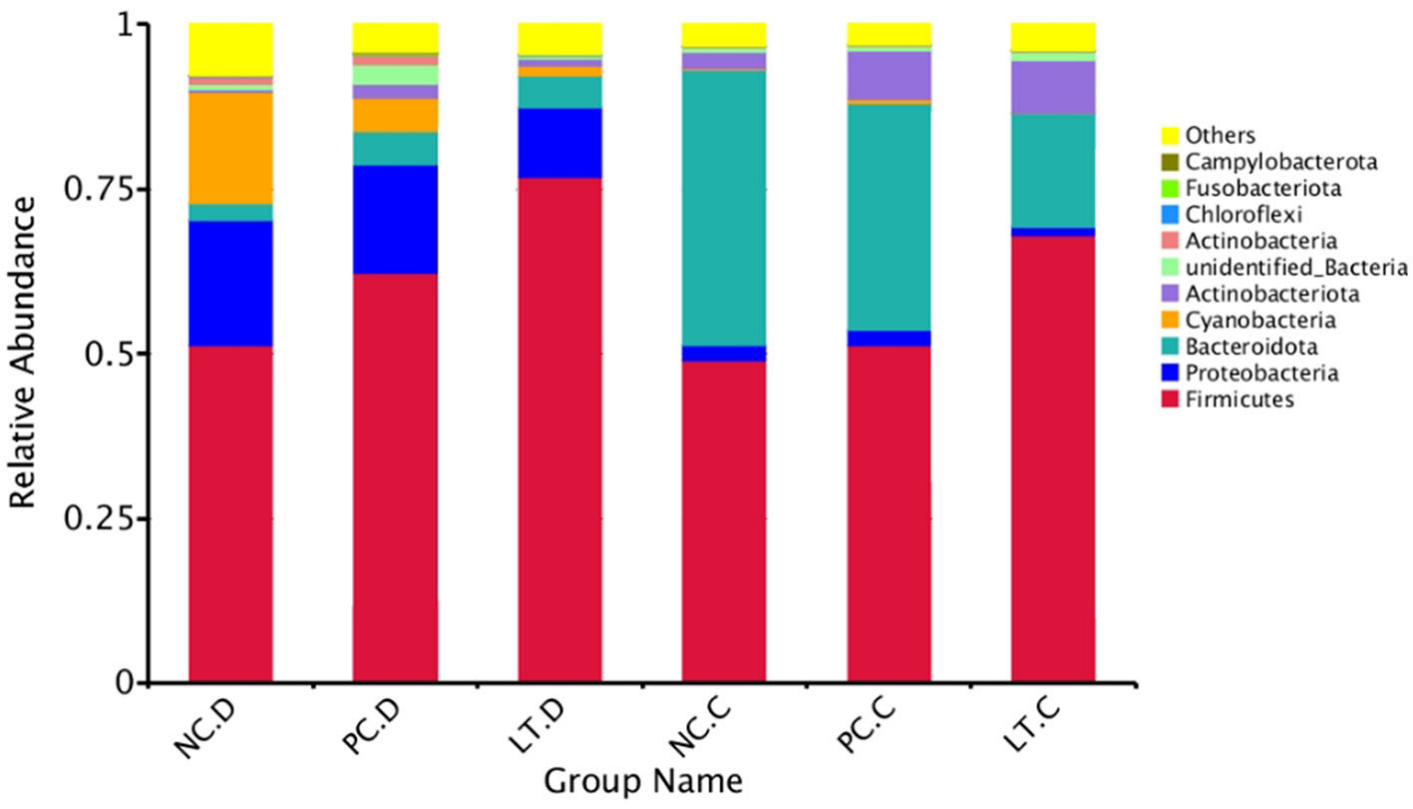
Histogram of phylum level species relative abundance. NC.D: the content of duodenum in NC group. PC.D, the content of duodenum in PC group. LT.D, the content of duodenum in LT group. NC.C, the content of cecum in NC group. PC.C, the content of cecum in PC group. LT.C, the content of cecum in LT group. ^a, b^Means within a row with different superscripts differ significantly (*P* < 0.05). Data represent the means of 6 bird for 1 bird per replicate (*n* = 6). PC, added hydrocortisone 30 mg/kg to drinking water in broilers basal diet; LT, fed diets supplemented with 400 mg/kg L-theanine to basal diets. NC, fed a corn-soybean meal basal diet.

According to the database annotation results, as shown in Figure 1, the top 10 functional pieces of information of each sample or group with the largest abundance at each annotation level were selected, and a columnar stacking bar was generated.

**Figure 2:**
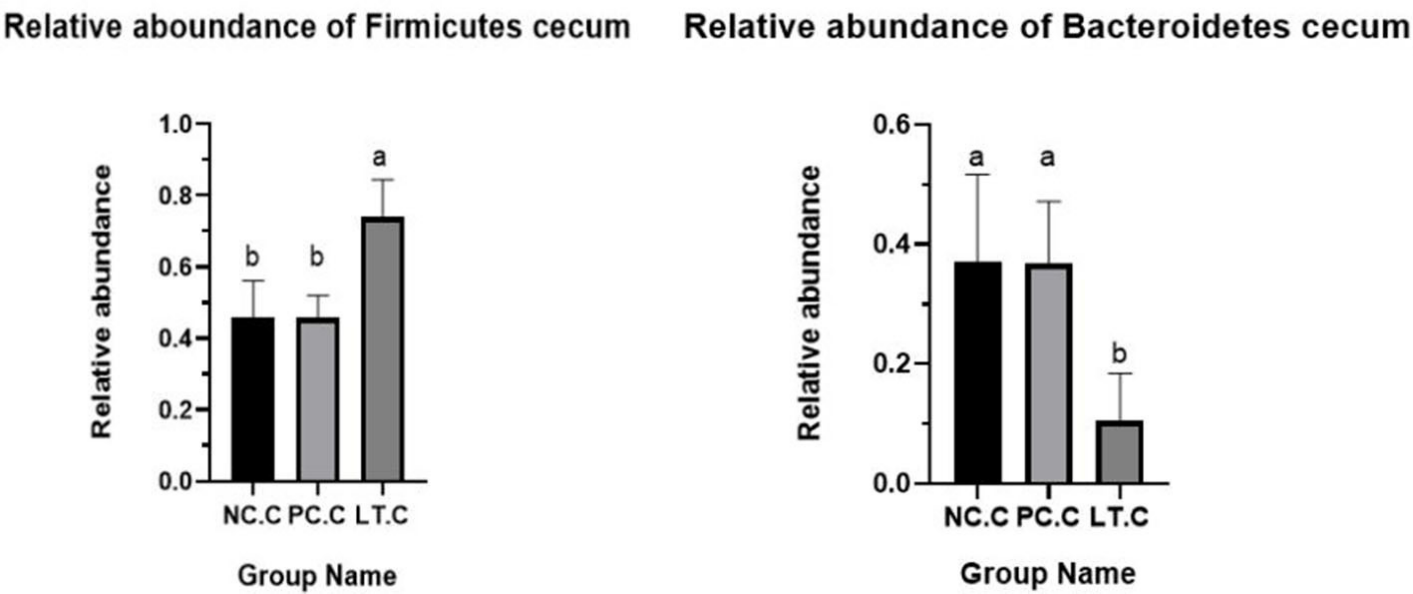
Abundance of firmicutes and bacteroidetes in cecum. NC.C, the content of cecum in NC group. PC.C, the content of cecum in PC group. LT.C, the content of cecum in LT group. ^a, b^Means within a row with different superscripts differ significantly (*P* < 0.05). Data represent the means of 6 bird for 1 bird per replicate (*n* = 6). PC, added hydrocortisone 30 mg/kg to drinking water in broilers basal diet; LT, fed diets supplemented with 400 mg/kg L-theanine to basal diets. NC, fed a corn-soybean meal basal diet.

**Figure 3:**
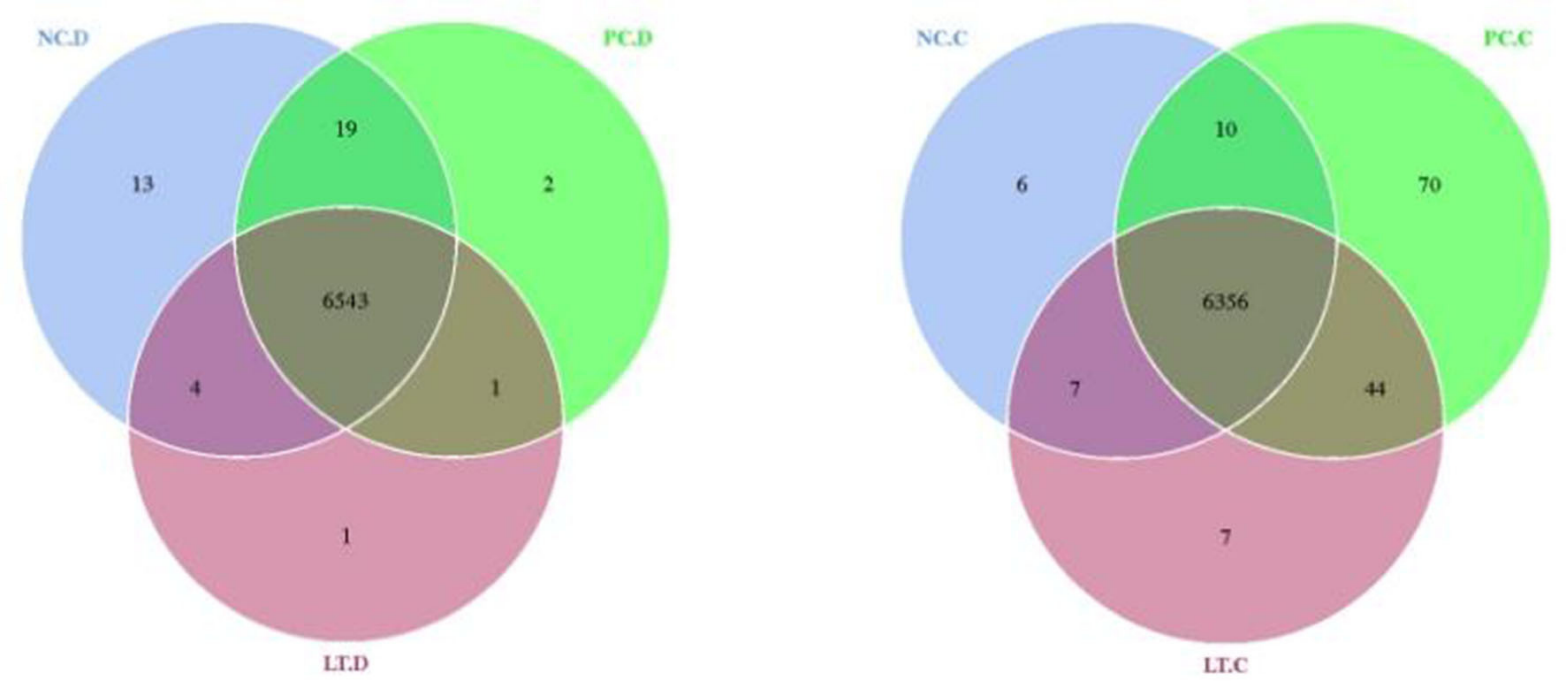
Venn diagram of OUT distribution between groups. NC.D, the content of duodenum in NC group. PC.D, the content of duodenum in PC group. LT.D, the content of duodenum in LT group. NC.C, the content of cecum in NC group. PC.C, the content of cecum in PC group. LT.C, the content of cecum in LT group. ^a, b^Means within a row with different superscripts differ significantly (*P* < 0.05). Data represent the means of 6 bird for 1 bird per replicate (*n* = 6). PC, added hydrocortisone 30 mg/kg to drinking water in broilers basal diet; LT, fed diets supplemented with 400 mg/kg L-theanine to basal diets. NC, fed a corn-soybean meal basal diet.

Figure 2 shows that the *Firmicutes* abundance of the LT group is significantly higher than the NC and PC groups at 42 days of age (*p* < 0.05). Figure 3 suggests that the *Bacteroidetes* abundance of the LT group is lower than the PC and NC groups (*p* < 0.05).

As shown in Figure 3, 6,379, 6,480, and 6,414 Operational Taxonomic Units (OTUs) are obtained from the cecal samples from NC, PC, and LT groups, respectively. The number of OTUs shared by the three groups was 6,356 accounting for 99.63, 98.80, and 99.09%, respectively. In total, 6,579, 6,565, and 6,549 OTUs were obtained from the duodenal contents of the NC, PC, and LT groups, respectively. The number of OTUs shared by the three groups was 6,543, accounting for 99.45, 99.66, and 99.91% separately.

## Discussion

Nowadays, oxidative stress is one of the research hotspots in the animal husbandry field. Under the state of oxidative stress, broilers have many problems, such as decreased growth performance and poor meat quality, which would affect economic benefits (16). Some studies have shown that it is an effective way to alleviate oxidative stress damage of animals by adding additives with antioxidant biological function (8). L-theanine is a natural plant extracted from tea with a powerful antioxidant capacity. In addition, it has other functions and can replace antibiotics (9). Due to the actual production cost of L-theanine, this experiment investigated the effects of a low dosage of L-theanine in broilers with oxidative stress induced by hydrocortisone. The results showed that 30 mg/kg of hydrocortisone in drinking water was significantly decreased ADG, ADFI, and BW and increased in F/G as compared to the NC group. Meanwhile, the LT and PC groups had significantly increased MDA concentrations in serum and liver at 24 days of age. It meant that the challenged chickens were on a state of oxidative stress (17). The LT group had a significantly lower F/G at 21–42 days of age as compared to the PC group. This revealed that it can improve the production performance of broilers with L-theanine supplementation under the oxidative stress. Other research results also indicated that the addition of L-theanine to broiler diets can improve the production performance of poultry (18).

The intestinal tract is an important organ, which is mainly responsible for digestion and absorption (19). Villus height and crypt depth are commonly used to evaluate the functional status of the small intestine (20). The crypt is considered to be a producer of villus cells, and the lower depth of crypt indicates that the intestinal villi are in good condition (21). Lower crypt depth and higher villus height mean a higher ability of the intestine to absorb nutrients (22). Some studies have proved that the intestinal absorption of nutrients mainly depended on the intestinal villi, and the intestinal villi epithelial cells absorbed nutrients from the digestive tract into the blood. At present, it is believed that there is a positive correlation between intestinal absorption and villi height (23). Therefore, parameters of intestinal mucosal morphology can explain the differences in production performance to a certain extent (24). The results of this study demonstrated that the villi height of jejunum in the PC group was significantly lower than in the NC group. This proved that oxidative stress has negative effects on the morphology of intestinal mucosa (25). However, no significant difference in villi height of jejunum between the LT and NC groups was observed. It suggested the broilers in diet with L-theanine could improve villi height of jejunum under oxidative stress. Meanwhile, the crypt depth of jejunum, ileum, and duodenum in LT group was declined than in the PC and NC groups. Some studies provided support to improve small intestinal morphology by feeding with L-theanine in poultry diets (26), which is similar to the results in our experiment. Hence, dietary L-theanine can improve intestinal mucosal morphology, so as to improve the production performance of broilers under oxidative stress.

More and more studies have demonstrated that the intestinal flora is closely related to the host (27). In addition, the number and type of intestinal flora can affect the nutrient absorption (28), health, and even behavior (29) of the host. Therefore, 16s rRNA analysis was performed to study the effects of L-theanine on the gut flora of broilers under oxidative stress. The results showed that oxidative stress decreased the number of specific genes in the duodenum of broilers, but supplementation of L-theanine did not improve this situation. This may indicate that L-theanine cannot improve the intestinal bacterial species abundance broilers under oxidative stress in this experiment. Studies have shown that L-theanine can affect the composition of gut microbiota (30). Compared with the NC and PC groups, the LT group had significantly increased the proportion of *Bacteroides* and *Firmicutes*. It proved that L-theanine could improve the intestinal flora composition. The intestinal tract was mainly dominated by *Firmicutes*, followed by *Proteobacteria* and *Bacteroidetes* (31). This is the same as our results. At the same time, the relative abundance of the *Firmicutes* in the LT group was significantly higher than the NC and PC groups. *Firmicutes* were considered to be able to resist harsh environments by their physiological function (32). Related studies have revealed that increasing *Firmicutes* bacteria is beneficial to intestinal health and can boost the production performance of broilers (33). Studies have shown that obesity is associated with a reduction in the *Bacteroidetes* and the ratio of *Firmicutes* to *Bacteroidetes* (34). In this experiment, the relative abundance of *Bacteroides* cecum was significantly lower than that in the NC and PC groups. It suggested that L-theanine can improve the production performance *via* modulating the composition of intestinal microflora.

The intestinal mucosal barrier is a structure, which can prevent harmful factors in the intestine from entering the lamina propria of the intestinal mucosa (35). It is an important mechanical protection for the internal and external environment of the body. Studies have reported that oxidative stress can damage the intestinal barrier function and further allow harmful factors in the intestinal tract to enter the body's internal environment, aggravating oxidative stress, and forming a vicious circle (36). Therefore, the integrity of gut barrier was important to relieve oxidative stress. ZO-1 protein is the earliest discovered intestinal tight junction protein. Some research studies have shown that the expression level of tight junction protein is positively correlated with barrier function (37). The ZO-1 activity of the LT group was significantly increased as compared to the PC and NC groups in this study. This suggested that L-theanine can improve the intestinal barrier.

Oxidative stress can damage cell by producing excess reactive oxygen species (ROS), which affects animal health (38). There are many findings that showed that dietary supplementation of natural antioxidants can eliminate ROS and improve oxidative damage and production performance (39, 40). Zhang et al. (41) found that L-theanine can ameliorate oxidative stress caused by transportation in animals. In this study, the concentrations of MDA, SOD, T-AOC, and GSH-Px in serum and liver were measured to investigate the effects of L-theanine on the antioxidant capacity of broilers under oxidative stress. The results showed that L-theanine significantly increased the GSH-Px activity in the liver of 42-days-old broilers, indicating that L-theanine has the effect of enhancing antioxidant capacity. It may partially explain why L-theanine can improve jejunum morphology. It should be noted that the results of other researchers suggested that L-theanine can not only increase the activity of GSH-Px but also improve other antioxidant indicators, such as T-AOC and SOD (42). In this experiment, only the activity of GSH-Px was increased. This might be due to the low dosage used in our study. People have increasingly focused on meat quality recently. Not only the meat quality is required to be safe, hygienic, and nutritious but also the meat color freshness by eyes and good taste was required. Previous research showed that shear force, PH value, and cooking loss can affect the taste of meat (43). Compared with the PC group, the PH was increased and shear force and cooking loss were decreased in LT group, which was consistent with other studies (41, 44). This indicated that L-theanine can improve meat quality, which may be related to the improvement of antioxidant capacity of oxidative stress broilers.

Adding 30 mg/kg of hydrocortisone to drinking water can significantly increase the MDA concentrations and decrease the F/G and ADG in the serum and liver. This experiment verified the feasibility of using hydrocortisone to establish an acute oxidative stress model in broilers. Meanwhile, dietary supplementation with 400 mg/kg of L-theanine from days 21, the results of this study proved that L-theanine can improve the F/G ratio under acute oxidative stress in broilers. F/G ratio is a key indicator to measure the performance of broilers. This suggested that L-theanine had positive effects on the production performance of the chicken under oxidative stress. It provides certain theoretical guidance for practical production.

## Conclusion

In conclusion, this study demonstrated that dietary supplementation of 400 mg/kg L-theanine could improve the production performance of broilers under oxidative stress. This may be achieved *via* improving gut morphology, enhancing antioxidant capacity, and modulating the intestinal microflora.

## Data Availability Statement

The data presented in the study are deposited in the NCBI repository, accession number PRJNA814510, https://www.ncbi.nlm.nih.gov/search/all/?term=PRJNA814510.

## Ethics Statement

The animal study was reviewed and approved by Animal Care and Use Committee of Foshan University.

## Author Contributions

ZW and YT trial conduction, data analysis, and draft of the manuscript. LL and HZ draft and revision of the protocol, revision of the manuscript, and final approval for paper publication. All authors contributed to the article and approved the submitted version.

## Funding

This work was supported by Discipline Construction Program of Foshan University (CGZ0400162), the Teachers' Characteristic Innovation Research Project of Guangdong University Research Findings Commercialization Center in Guangdong Province, China (2020SWYY02), the Open fund of Key Laboratory of Animal Nutritional Physiology and Metabolic Processes of Hunan Province, Institute of Subtropical Agriculture, Chinese Academy of Sciences in Hunan Province, China (ISA2020112), and the Scientific Research Foundation in the Higher Education Institutions of Educational Commission of Guangdong Province (2017GCZX006).

## Conflict of Interest

The authors declare that the research was conducted in the absence of any commercial or financial relationships that could be construed as a potential conflict of interest.

## Publisher's Note

All claims expressed in this article are solely those of the authors and do not necessarily represent those of their affiliated organizations, or those of the publisher, the editors and the reviewers. Any product that may be evaluated in this article, or claim that may be made by its manufacturer, is not guaranteed or endorsed by the publisher.
